# Nitrogen and oxygen Co-doped porous carbon derived from yam waste for high-performance supercapacitors†

**DOI:** 10.1039/d1ra06154b

**Published:** 2021-10-08

**Authors:** Zhaojin Li, Qian Liu, Lizhi Sun, Ning Li, Xiaofeng Wang, Qiujun Wang, Di Zhang, Bo Wang

**Affiliations:** Hebei Key Laboratory of Flexible Functional Materials, School of Materials Science and Engineering, Hebei University of Science and Technology Hebei 050018 China wangbo1996@gmail.com zd1210207@163.com; Shenzhou Engineering Plastics Company Limited Boling East Road 106, Shenzhou Economic Development Zone Hebei 053800 China

## Abstract

It is a considerable challenge to produce a supercapacitor with inexpensive raw materials and employ a simple process to obtain carbon materials with a high specific surface area, rich pore structure, and appropriate doping of heterogeneous elements. In the current study, yam waste-derived porous carbon was synthesized for the first time by a two-step carbonization and KOH chemical activation process. An ultra-high specific surface area of 2382 m^2^ g^−1^ with a pore volume of 1.11 cm^3^ g^−1^ and simultaneous co-doping of O–N was achieved for the optimized sample. Because of these distinct features, the optimized material exhibits a high gravimetric capacitance of 423.23 F g^−1^ at 0.5 A g^−1^ with an impressive rate capability at 10 A g^−1^, and prominent cycling durability with a capacity retention of 96.4% at a high current density of 10 A g^−1^ after 10 000 cycles in 6 M KOH in a three-electrode system. Moreover, in 6 M KOH electrolyte, the assembled symmetrical supercapacitor provides a large *C* of 387.3 F g^−1^ at 0.5 A g^−1^. It also presents high specific energy of 34.6 W h kg^−1^ when the specific power is 200.1 W kg^−1^ and a praiseworthy specific energy of 8.3 W h kg^−1^ when the specific power is 4000.0 W kg^−1^ in 1 M Na_2_SO_4_ electrolyte. Thus, this study provides reference and guidance for developing high-performance electrode materials for supercapacitors.

## Abstract 2

3D porous carbon with ultra-high specific surface area and excellent electrochemical performance is synthesized by a simple activation and carbonization process through adopting biomass yam waste as raw material.

## Graphical abstract

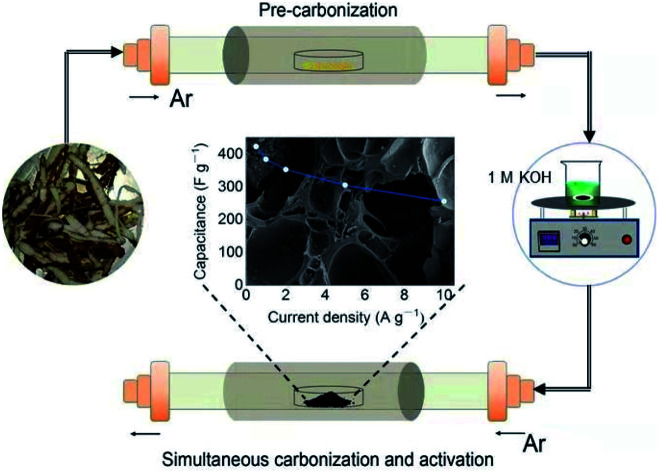

## 1. Introduction

With the development and application of portable electronic devices and electric vehicles, there is considerable demand for efficient energy storage devices.^1,2^ Particularly, supercapacitors have great potential in this regard because of their long cycle life, high efficiency, and outstanding power density.^3–5^ In terms of mechanism, there are two types of supercapacitors: (i) an electric double-layer capacitor (EDLC) that stores charge at the electrode/electrolyte interface, and (ii) a pseudo capacitor (PC) related to a reversible Faraday redox reaction. Of the two, the EDLC has incomparable advantages in terms of cost, cycle performance, and safety.^6,7^ Therefore, the commercial supercapacitors currently being manufactured are mainly EDLCs.^8^

The energy storage capacity of EDLCs is derived from the adsorption/desorption of electrolyte ions at the interface between the active material and electrolyte. The specific surface area (SSA) of the active material holds the key to capacity performance. In fact, the SSA is not the only factor determining the capacitance of EDLCs.^9^ Pore feature, conductivity, and heterogeneous element doping are also significant parameters influencing the electrochemical performance of porous carbon materials.^10^ In the past decade, researchers have attempted to enhance the electrochemical properties of EDLCs through the following methods: (i) enlarging the SSA of active sites;^11–13^ (ii) designing new nanostructures for shortening ion diffusion paths;^14,15^ (iii) designing more appropriate porosity (interwoven micropores, mesopores, and macropores) for achieving fast ion transport and improving rate capability;^16,17^ and (iv) introducing defects and/or heteroatoms to increase pseudocapacitance and improve their electronic and/or chemical properties.^18,19^

As for electrode materials, there are many types of carbon materials that can be used as active materials for supercapacitors, such as graphene, carbon nanotubes, and activated carbon. Biomass carbon materials are considered as potential active materials because they feature high yields, a natural construction that can regulate the pore structure, and are environmentally friendly. Biomass precursors are advantageous because they can directly introduce a large number of heteroatoms. Moreover, biomass carbon materials often contain adjustable heterogeneous elements such as N, O, S, B, and P.^20–24^ These heteroatoms induce additional electrochemical active sites to generate a positive effect on the capacity of the supercapacitor, and they also increase the surface wettability to obtain a stronger rate performance.^7,25^

Based on the aforementioned advantages, it is very environmentally friendly to utilize biomass as a raw material to construct 3D porous carbon with high SSA, high conductivity, and heteroatom doping for achieving excellent electrochemical performance in supercapacitors. Thus, numerous biomass-derived carbon materials (such as fish scales,^9^ argan seed shells,^26^ bio-oil,^27^ banana peels,^28^ soybean dregs,^10^ oil-tea seed shells,^29^ and bamboo leaves^30^) have been widely reported.

Biomass precursors are generally classified into several categories: cellulose,^31^ lignin,^32^ chitin,^33,34^ starch,^35^ and protein.^36^ Among them, starch with a general formula of (C_6_H_10_O_5_)_*n*_ is abundant and inexpensive. When carbonization is carried out, two-fold H and one-fold O atoms in the precursor combine to form water and escape, leaving biomass carbon materials. In the process, vast pores can be generated. Thus, the activated carbon not only exhibits satisfactory conductivity but it is also rich in micropores, which can meet the requirements of electrode materials for EDLCs. Thus, a large amount of starch-rich biomass has been considered for use as a material from which to construct electrodes for supercapacitors.

For instance, Zhong *et al.*^37^ synthesized starch-derived porous carbon using the sol–gel method, which exhibited a specific capacitance of 272 F g^−1^ and a current density of 1 A g^−1^. Wu *et al.*^38^ prepared microporous carbon using potatoes as the carbon source under different processing conditions, and obtained the highest specific capacitance of 337 F g^−1^ at 1.0 A g^−1^ in a three-electrode system. Compared with this research, yams also contain a massive amount of starch and may exhibit more optimal electrochemical properties. However, the fabrication of biomass carbon by employing yams as a precursor has not been fully studied.

Hence, in this work, a facile and effective carbonization–activation method for the preparation of 3D hierarchical porous carbon using yam waste as a precursor is reported for the first time. The as-prepared yam porous carbon (YPC) possesses an ultra-high SSA of 2382 m^2^ g^−1^ and maintains an appropriate heteroatom content. Because of these unique features, YPC exhibited proven electrochemical properties with a large weight capacitance of 423.23 F g^−1^ at a current density of 0.5 A g^−1^. After 10 000 cycles in 6 M KOH electrolyte, the capacity retention rate at 10 A g^−1^ was 96.4%. Additionally, the assembled symmetrical supercapacitor provided a large specific capacitance of 387.3 F g^−1^ in the 6 M KOH electrolyte at 0.5 A g^−1^. Moreover, in 1 M Na_2_SO_4_ electrolyte, the assembled symmetrical supercapacitor presented a large energy density of 34.25 W h kg^−1^ when the power density was 200.13 W kg^−1^, and remained at 8.3 W h kg^−1^ even at a rather large power of 4000 W kg^−1^. The method presented in this study can serve as a reference for synthesizing other carbonaceous materials originating from starch biomass, and the resulting products can simultaneously fulfill considerable rate capability and capacity.

## 2. Materials and methods

### 2.1 Materials

Yam waste was collected from a local supermarket, washed in DI water three times, dried at 70 °C for 24 h, and ground into a powder by ball milling for 30 min at 200 rpm. All chemical reagents were analytical grade and were used without any further purification.

### 2.2 Synthesis of YPC

**Fig. 1:**
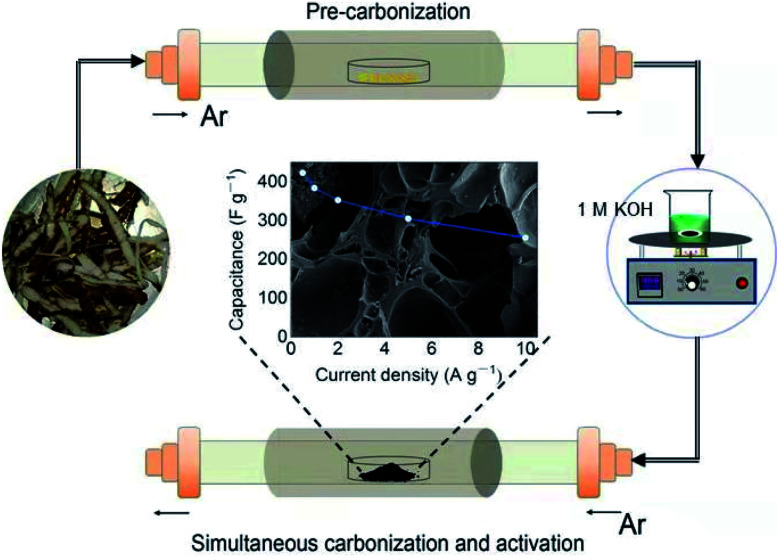
Schematic illustration of YPC derived from yam waste.

The preparation process of YPC is shown in Fig. 1. First, the yam waste powder was placed into a corundum boat and pre-carbonized at 450 °C for three hours under an argon atmosphere at a heating rate of 1 °C min^−1^. Subsequently, the pre-carbonized samples were chemically activated with KOH under argon flow at different temperatures (600–800 °C) for 3 hours to prevent the pre-carbonized samples from being oxidized. The activation process is as follows: 1.5 g yam waste and 53.5 mL 1 M KOH (3.0 g KOH) were stirred at 60 °C until the liquid was almost completely evaporated, and then, the mixture was dried in a drying oven at 70 °C overnight. The resulting product was named YPC-*T* (*T* = 600, 700, 800), where *T* (600, 700, 800) indicates the carbonization temperature.

After the optimum temperature was obtained, the pre-carbonization product was mixed with 1 M KOH at the mass ratio of 1 : 2, 1 : 3, 1 : 4, and 1 : 5, respectively. Then, the products with KOH were heat-treated at different temperatures using the same method as that described above for the waste yams. Finally, the washed carbonizing samples were placed into a drying cabinet at 70 °C overnight to obtain the porous carbon products named YPC-*n* (*n* = 2, 3, 4, 5), where *n* (2, 3, 4, 5) indicates the ratio of the KOH to the pre-carbonized products.

### 2.3 Characterization

Thermogravimetry analysis (TG, Setaram, Setsys 16-18) was conducted at a heating rate of 5 °C min^−1^ in a high purity argon atmosphere. Morphological studies of the YPC were performed by scanning electron microscopy (SEM, Zeiss Sigma 500). Samples for TEM and high-resolution TEM (HRTEM, FEI Tecnai G2 F20, USA) analysis were prepared by drying a drop of materials dispersed in alcohol on amorphous carbon-coated copper grids, and the microscope was operated at 200 kV. X-ray powder diffraction patterns were obtained using a Bruker D8 Advance X-ray powder diffractometer. Unpolarized Raman spectra were collected to analyze the lattice defects of the material using a Raman spectrometer (HORIBA JY LabRAN HR Evolution).

The elemental composition of the materials was evaluated by X-ray photoelectron spectroscopy (XPS, Escalab 250). The Brunauer–Emmett–Teller (BET) surface area (Micromeritics ASAP 2020, USA) was calculated from the linear plots in the relative pressure range within 0.05–0.35, and the pore size distribution was calculated according to the adsorption branch of the isotherm using the non-local density functional theory (NLDFT) model. The amounts of organic elements of C, H, N, and O in the YPC-4 sample were detected by an elemental analyzer (EA, Elementar Vario Microcube). Inductively coupled plasma-optical emission spectrometry (ICP-OES, Varian 720-ES) was used to determine the chemical composition of the ash content in the YPC-4 sample.

### 2.4 Electrochemical tests

The electrochemical properties of YPC were evaluated employing a three-electrode system. For the preparation of the test electrode, the YPC and polytetrafluoroethylene were dispersed in deionized water with a mass ratio of 9 : 1 and stirred until it appeared spongy. Subsequently, the slurry was combined with two pieces of foamed nickel (1.5 × 1.5 cm^2^) to form a sandwich electrode. The loading of active materials on the electrode was approximately 3 mg cm^−2^. The as-prepared electrode, Hg/HgO, platinum foil (2 × 2 cm^2^), and 6 M KOH were used as the working electrode, reference electrode, counter electrode, and electrolyte, respectively.

Galvanostatic charge–discharge (GCD), cyclic voltammetry (CV), and impedance measurement experiments were performed using an electrochemical working station (Princeton). On the basis of the GCD curves, in a three-electrode system, the specific capacity of a single electrode was calculated by using eqn (1):1
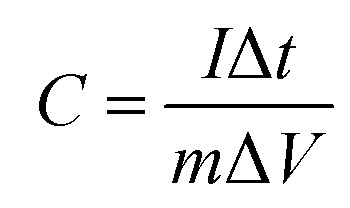
where *C* represents the specific capacitance (F g^−1^), *I* (A) represents the discharge current, Δ*t* (s) represents the discharge time, *m* (g) represents the mass of the active material, and Δ*V* (V) represents the voltage window in discharge.

In addition to the three-electrode system, a symmetric supercapacitor was assembled using YPC as the anode and cathode to investigate the related electrochemical performance in 6 M KOH and 1 M Na_2_SO_4_ aqueous solutions, separately. Based on the GCD curve, the *C* of a single electrode (*C*_s_) and the total cell (*C*_cell_) can be well defined through the following eqn (2) and (3):2
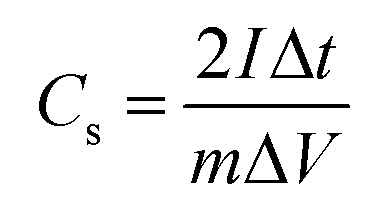
3
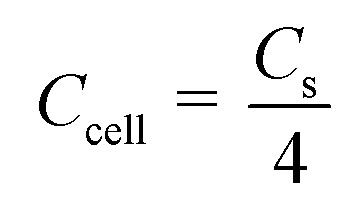
where *I* (A) represents the discharge current, Δ*t* (s) represents the discharge time, *m* (g) represents the mass of the active material of each electrode, and Δ*V* (V) represents the voltage window in discharge.

The energy density (*E*) and power density (*P*) of the double-electrode symmetric supercapacitor are defined by eqn (4) and (5):4
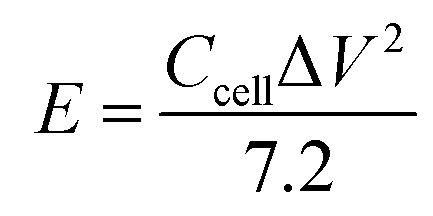
5
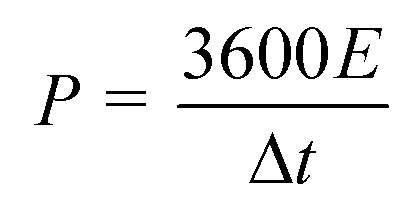
where Δ*V* (V) indicates the voltage window, and Δ*t* (s) represents the discharge time.

## 3. Results and discussion

### 3.1 Characterization of the YPC

Fig. S1† shows the TG curve of the yam waste precursor, and it indicates that mass loss for the biomass precursor of the whole heat treatment was approximately 85%, mainly in the pre-carbonization process. The slight mass loss at temperatures below 250 °C resulted from free water loss in the precursor. Significantly, the major weight loss occurred in the range of 250–450 °C, and resulted from bound water loss and organic matter decomposition. Thus, the precursor was first pyrolyzed at 450 °C to avoid deliquescence and maintain a durable microstructure.^39,40^

**Fig. 2:**
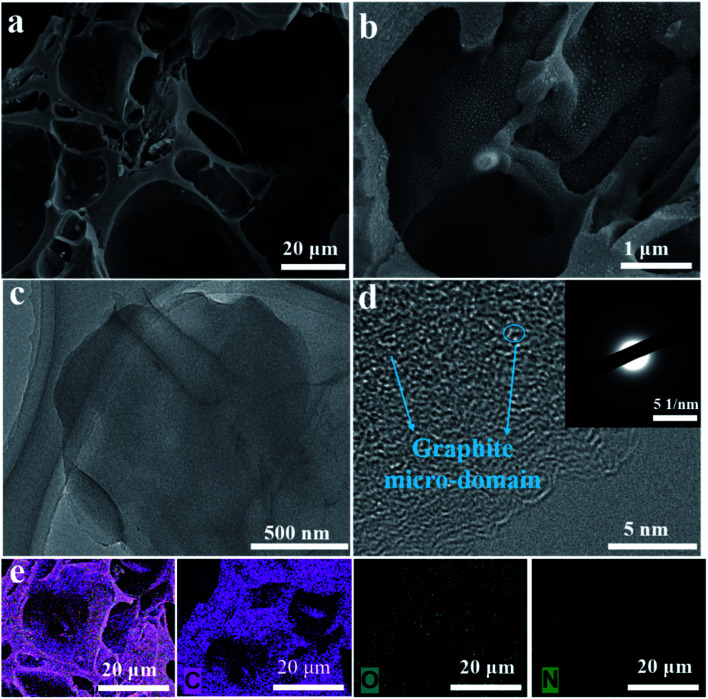
Morphology of the YPC-4 sample after activation. (a, b) SEM images of the YPC-4, (c, d) TEM images of the YPC-4, and (e) EDX elemental mapping images of YPC-4.

To explore the morphology and structure of the pre-carbonized and subsequent KOH-activated YPC samples, SEM and TEM were used. The morphologies of the YPC-4 show a pseudo-honeycomb-like 3D net structure with a pore diameter of approximately 50 μm and a thickness of 1–10 μm (Fig. 2a and b). This structure is conducive to the efficient diffusion of ions when the prepared YPC-4 samples are used as an electrode material for a supercapacitor. However, a sample without activation assumes an irregular lump microstructure without any pores in it (Fig. S2a†).

The pre-carbonized samples were activated by different amounts of KOH with similar porosity characteristics (Fig. S2b–d†), which are helpful for the diffusion of electrolyte ions into the internal micropores. Therefore, we know that macropores are formed by the etching effect of biomass precursors by KOH. The activated carbon materials of YPC-4 were further characterized by TEM (Fig. 2c), which detected a thin sheet morphology existing in YPC-4 samples. The HRTEM image shows that the synthesized YPC-4 samples are mainly amorphous carbon, and this result can be further verified by the selected electron diffraction pattern (inset in Fig. 2d). Typical energy-dispersive X-ray (EDX) spectra demonstrate that the YPC-4 sample is composed of C, O, and N elements (Fig. 2e), with uniform distribution of O and N in the carbon matrix.

**Fig. 3:**
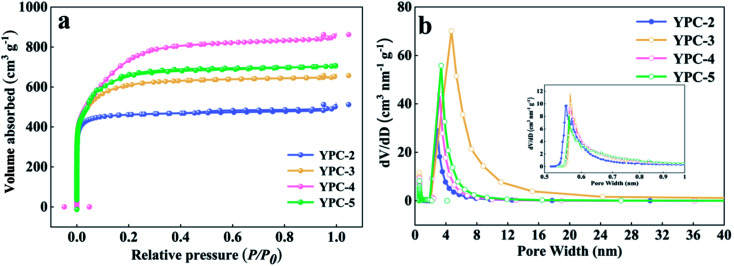
(a) N_2_ adsorption–desorption isotherms, (b) plots of pore size distribution of the YPC-2, YPC-3, YPC-4, and YPC-5 samples.

Further information on the SSA and pore structure of the YPC samples was acquired from N_2_ adsorption–desorption isotherm measurements (Fig. 3a and b and S3b†). All the adsorption of the YPC-*n* samples occurred at a relatively low pressure (*i.e.*, *P*/*P*_0_ < 0.04) and reached a stable state at high relative pressures, suggesting the existence of micropores.^4^ When the value of *P*/*P*_0_ is close to 1, the curve exhibits a slight upward trend, which may be due to adsorbate condensation. As depicted in Table 1, the SSA of the YPC samples was greatly increased by the KOH treatment. Additionally, the porosities of the carbon materials are dependent upon the quantity of KOH. Moreover, the YPC-4 sample with the mass ratio (KOH/pre-carbonized yam waste) of 4 : 1 exhibited the highest SSA of 2382 m^2^ g^−1^ with a relatively high pore volume of 1.11 cm^3^ g^−1^ compared with the other samples. As seen in Fig. 3b, there are two sharp peaks in the pore size distribution plot of the YPC-4 samples, which are located at 0.56 and 3.37. What is noteworthy is that the ultra-high SSA of the YPC-*n* samples can provide numerous sites for ion adsorption. Additionally, exposure of these high porosity YPC-*n* nanosheets to electrolyte can effectively shorten the ion diffusion path, reduce the ion diffusion resistance, and eventually lead to a stronger rate performance when they are used as electrode materials for supercapacitors.

**Table 1:** Pore structural parameters and compositions of the samples

Sample	*S*_BET_ (m^2^ g^−1^)	*V*_total_ (m^3^ g^−1^)	*V*_micro_ (m^3^ g^−1^)	C (%)	O (%)	N (%)
YPC-2	1855	0.85	0.72	85.46	12.56	1.3
YPC-3	2226	1.01	0.93	89.09	9.35	1.56
YPC-4	2382	1.11	1.02	91.38	7.82	0.8
YPC-5	2320	1.33	1.12	86.82	12.72	—

**Fig. 4:**
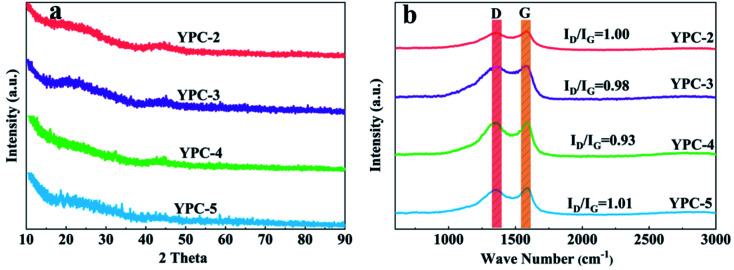
(a) XRD patterns and (b) Raman spectra of the YPC-2, YPC-3, YPC-4, and YPC-5 samples.

The crystallinity of the YPC-*n* samples was examined by XRD analysis (Fig. 4a and S3a†). The broad diffraction peaks centered at 25° and 43° correspond to the (002) and (100) diffraction of the disordered carbon layer, respectively. There is a low graphitization degree for the synthesized YPC-*n* samples, which is consistent with the HRTEM and SAED results. By comparison, the XRD patterns of YPC-4 indicate a higher intensity of the (002) peak in comparison with the other samples, confirming an increased degree of graphitization. Moreover, it is noteworthy that the intensity of the YPC-*n* samples increases at the low-angle scattering peak. As reported by Zhu *et al.*, the strong X-ray diffraction peaks in the low-angle region indicate that there are a large number of micropores in the YPC-*n* samples, which is consistent with the measurements of pore size distribution.^41^

The Raman spectra of the YPC-*n* samples in Fig. 4b show that there are two strong peaks near the 1350 cm^−1^ (D band) and 1590 cm^−1^ (G band) in all samples. The D band reflects the disordered and defective structures in carbon materials, while the G band is caused by the vibration of sp^2^-hybridized carbon in graphite crystal. Therefore, the graphitization degree of the synthesized carbon materials can be expressed by the ratio of the relative strength of the D peak to the G peak (*I*_D_/*I*_G_).^42,43^Fig. 4b shows that the *I*_D_/*I*_G_ ratio of YPC-2, YPC-3, YPC-4, and YPC-5 are estimated to be 1.00, 0.98, 0.93, and 1.01, respectively. Compared with other obtained carbon materials, the lowest *I*_D_/*I*_G_ value is for the YPC-4 sample, which indicates that it has a relatively high degree of graphitization degree and conductivity.

**Fig. 5:**
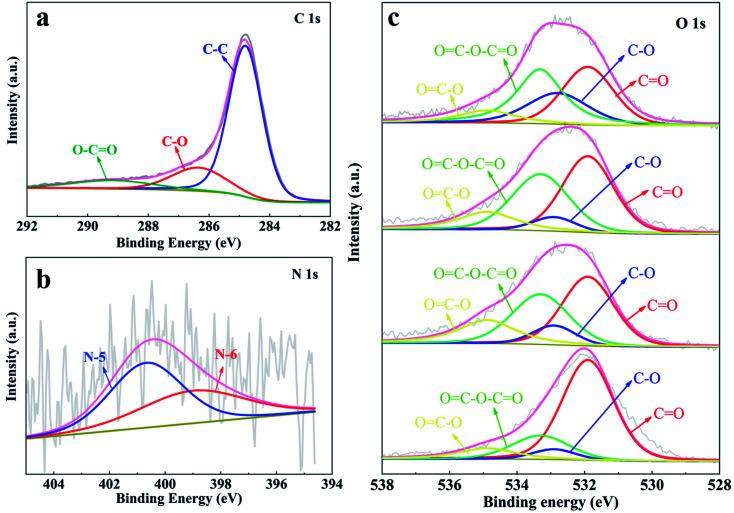
XPS spectra of (a) C 1s; (b) N 1s for YCP-4; and (c) O 1s for the YPC-2, YPC-3, YPC-4, and YPC-5 samples.

To evaluate the elemental composition and bonding state of the heteroatoms in the as-prepared YPC-*n*, XPS measurement was conducted. The detection outcomes show that all the synthesized samples contain C, O, and N elements (Fig. S4†). We further employed the Gaussian–Lorentzian curve-fitting method to fit the XPS spectra of C, O, and N 1s (Fig. 5). The spectra signal of C 1s showed that there was a main peak at 284.5 eV, which indicated that the YPC-*n* materials are mainly composed of sp^2^-hybridized C–C bonds.^44,45^ In addition, there were two small peaks at 286.3 eV and 289.2 eV, indicating that some C–O and O=C–O bonds exist in the carbon samples (Fig. 5a).^9,15^ The N 1s spectrum (Fig. 5b) showed that there were two forms of N, namely, N-5 (400.7 eV) and N-6 (400.0 eV), in the YPC-4 materials.^46^ As already observed, a large amount of O was detected in the YPC-*n* samples. By fitting the O 1s spectra, two main peaks located at 532.9 eV and 534.9 eV were clearly identified, which indicate the existence of C–O and O=C–O bonds in the samples (Fig. 5c). Two small peaks at 531.9 eV and 533.3 eV were also detected that indicate that there are some *C*=O and O=C–O–C=O bonds in the carbon samples.^47,48^

Considering that XPS shows only the detected information in the sample from the outermost surface, for an exact understanding of the chemical composition of the samples, EA and ICP tests were performed. The comprehensive analysis of the results from the EA (Table S1†) and ICP (Table S2†) tests showed that the elemental amounts of C, N, O, and ash (mainly including K and Al) are 44.89%, 0.33%, 46.24%, and 5.3%, respectively. These results further demonstrate that N and O elements were successfully doped into the interior of the YPC-4 sample.

It should be noted that the presence of N and O enhances the surface affinity of carbon to the electrolyte and produces pseudocapacitance, which increases the overall capacitance.^49^ Adding N positively affected the carbon conductivity because it provided more electron lone pairs. In addition, the N-doping produced more active sites for ion absorption.^50,51^ The O-doping enhanced the wettability of the electrode material so that the electrolyte ions can rapidly spread on the surface of the active material. Compared with single heteroatom doping, N and O co-doping enhanced the overall electrochemical properties of materials through a synergistic effect.^52,53^

### 3.2 Electrochemical properties in the three-electrode system

**Fig. 6:**
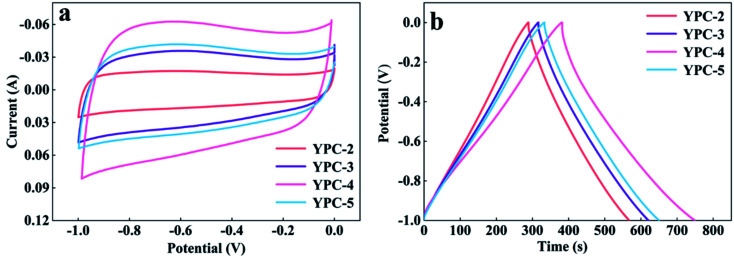
Electrochemical performance of the YPC-2, YPC-3, YPC-4, and YPC-5 samples in the three-electrode setup. (a) CV curves at 10 mV s^−1^; and (b) GCD profiles at 1 A g^−1^.

CV, GCD, and electrochemical impedance spectroscopy (EIS) techniques were applied to evaluate the electrochemical performance of the YPC-*n* samples in 6 M KOH in a three-electrode system at 25 °C. The CV profiles of the YPC-*n* electrodes (Fig. 6a and S5a†) were measured at scan rates of 10 mV s^−1^ between −1.0 and 0 V. As observed, the CV curves generally present a symmetrical rectangular shape, which indicates that the electrochemical reaction is dominated by the EDLC. In addition, we can see that the rectangles show obvious bulges, which are due to the pseudocapacitance caused by the O and N functional groups.^47^ The GCD curves of the YPC-*n* electrodes (Fig. 6b and S5b†) showed triangular symmetry. It was further proved that the main electrochemical behavior of the YPC-*n* electrodes is EDLC. Apparently, the YPC-4 exhibits a better capacitance performance than those of the YPC-2, YPC-3, and YPC-5, and this is mainly because the YPC-4 materials have a larger SSA and pore volume.

**Fig. 7:**
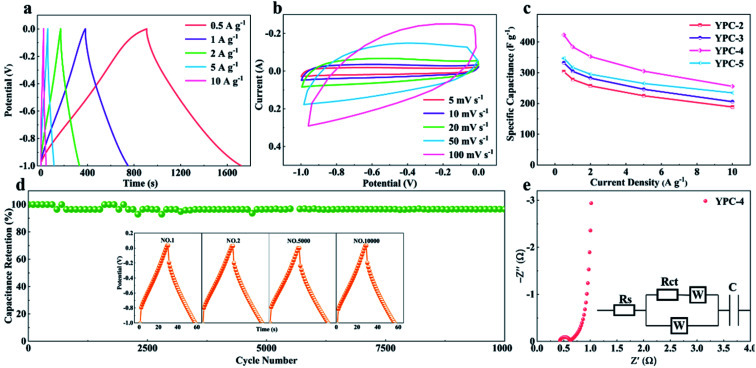
Electrochemical performance of YPC-4. (a) GCD profiles at different current densities; (b) CV curves at different scan rates; and (c) specific capacitance values of YPC-4 at different current densities. (d) Cycling performance of the YPC-4 electrode over 10 000 cycles at 10 A g^−1^; the inset shows the GCD profiles at different cycles. (e) Nyquist plot of the YPC-4 electrode; the inset shows the corresponding equivalent circuit model.

All the GCD curves of the YPC-4 electrode at various current densities show a similar triangular symmetry, indicating that the electrochemical behavior of the YPC-4 is mainly the EDLC mechanism. Fig. 7a. Fig. 7c shows that the specific capacitance of is 423.2, 384.2, 352.8, 305.3, and 255.9 F g^−1^ at current densities of 0.5, 1.0, 2.0, 5.0, and 10.0 A g^−1^ in 6 M KOH electrolyte, respectively. The cycling stability of the electrode was further studied through a GCD test at 10 A g^−1^. After 10 000 cycles, the specific capacitance of the YPC-4 electrode only decreased from 280 to 270 F g^−1^, showing that the synthesized carbon materials have an extraordinary cycle stability (Fig. 7d).

The inset of Fig. 7d shows that there is almost no change in the GCD curves under different cycles, which further confirms the cycling durability of the synthesized carbon materials. Such excellent electrochemical performance is closely related to ultra-high SSA, abundant micropores, and well-developed porous structure, which guarantees the fast ion transfer by reducing path distance upon charging and discharging. It is worth mentioning that the presence of ash has a negligible effect on the electrochemical performance of the porous carbon materials. This is because the measured specific capacity of the ash after removing all organic elements (C, O, N) from the YPC-4 is nearly 0 (Fig. S6†). Additionally, the ash content is very small, as detected from the ICP test (Table S2†).

The electron/ion transport properties of the YPC-4 electrode were further studied by EIS (Fig. 7e). The illustration shows the equivalent circuit simulation diagram. The abscissa of the intersection of the semicircle and the *x*-axis represents the total internal resistance (*R*_s_) of the electrode material. The fitting values of *R*_s_ and *R*_ct_ are 0.36 and 0.31 Ω, respectively, which indicates that the electrode has very low series resistance and charge transfer resistance. In the low frequency region, the EIS curve shows a vertical upward state, indicating that the electrode possesses strong capacitance characteristics and low ion diffusion resistance. The electrochemical properties of porous carbons prepared with different raw materials are compared in Table S3,† and the results demonstrate that the electrochemical performance of YPC-*n* electrodes is remarkable when compared with the reported literature.

### 3.3 Electrochemical properties of the two-electrode system

**Fig. 8:**
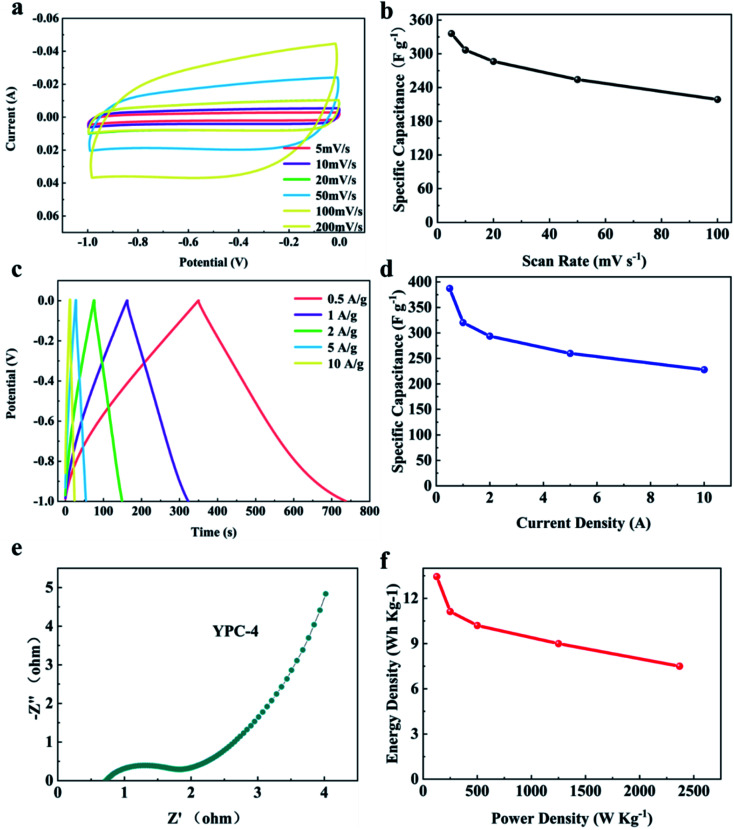
The symmetrical two-electrode test in 6 M KOH electrolyte. (a) CV profiles at different scan rates; (b) specific capacity performance values at different scan rates; (c) GCD profiles at different current densities; (d) the specific capacity performance at different current densities; (e) Nyquist plot of the YPC-4 monolithic electrode; and (f) the specific energy and power analysis of YPC-4 in the two-electrode system.

For evaluating the practical application of the synthesized electrode materials, two YPC-4 electrodes were employed for constructing a symmetrical supercapacitor in 6 M KOH aqueous electrolyte. Fig. 8a shows that at various scanning rates of 5 to 100 mV s^−1^, all CV curves were rectangular, demonstrating a typical double-layer capacitance behavior. Particularly, the specific capacitance of YPC-4 can reach 373 F g^−1^ at a scanning rate of 5 mV s^−1^ (Fig. 8b).

The GCD curves showed symmetrical triangle shapes, implying typical electrical double-layer capacitor performance for the YPC-4 sample, which is in perfect agreement with the CV outcome (Fig. 8c). The *C* values at various current densities were computed from GCD curves and are summarized in Fig. 8d, and show that the discharge time was 387.3 s at 0.5 A g^−1^, and the calculated *C* value of a single electrode was 387.3 F g^−1^ at 0.5 A g^−1^. When the current density increased to 10 A g^−1^, a capacitance retention of 58.9% (228 F g^−1^) was achieved, indicating a satisfactory rate performance. Nevertheless, in a symmetrical battery, the *C* value of a single electrode computed at a given current density was slightly below that of the working electrode in a three-electrode system.

There are two possible causes for this phenomenon: (a) the two-electrode system is an array of systems composed of two YPC electrodes separated by the separator, and its *R*_s_ exceeds that of the three-electrode system, causing higher energy consumption; (b) in the three-electrode system, the working potential of the YPC-4 electrode is associated with the Hg/HGO electrode (an almost ideal reversible electrode). Nevertheless, in the practice of a two-electrode system, one of the electrodes was employed as a reference electrode. Therefore, the working potential deviated from the set potential.

Fig. 8e displays the Nyquist plot for the YPC-4 monolithic electrode. The small radius of the semicircle in the high frequency region suggests that *R*_ct_ is very low, and the large slope at the low frequency region implies that there is double-layer capacitance behavior of the YPC-4 electrode. Fig. 8f shows that the energy density of the YPC-4 electrode was calculated as 13.45 W h kg^−1^ when the power density was 125 W kg^−1^, and it can retain 7.5 W h kg^−1^ as the power density increased to 2368.4 W kg^−1^.

**Fig. 9:**
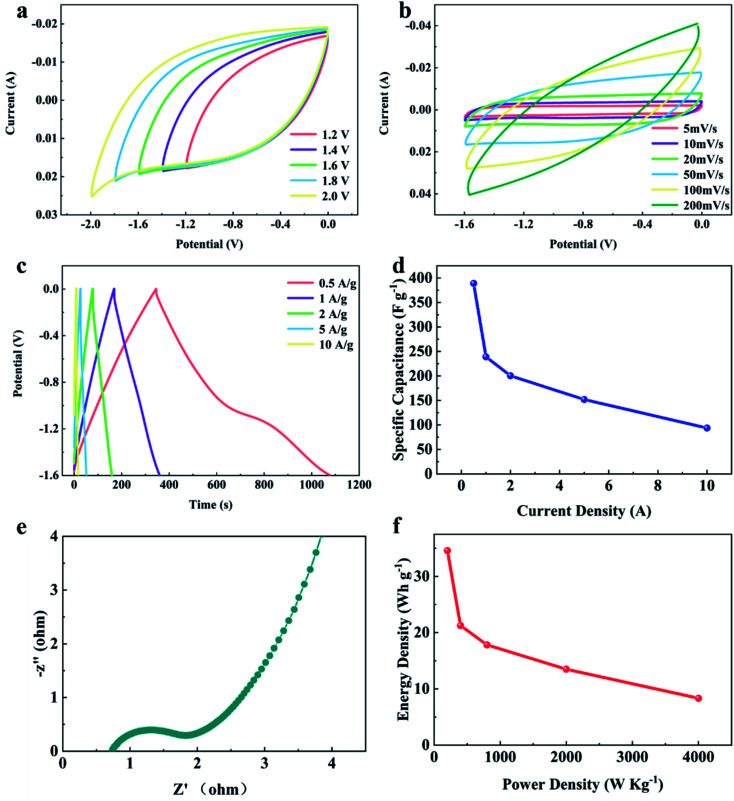
Electrochemical performance of YPC-4 in the two-electrode system with 1 M Na_2_SO_4_ as electrolyte. (a) CV curves at different operating voltages from −1.2–0 to −2.0-0V with a scan rate of 50 mV s^−1^; (b) CV curves at different scan rates; (c) GCD profiles at different current densities; (d) the specific capacity performance at different current densities; (e) Nyquist plot of the YPC-4 electrode; and (f) the specific energy and power analysis of the YPC-4 in the two-electrode system.

The influence of electrolyte type was further investigated. To evaluate the same symmetrical supercapacitor with the YPC-4 electrode, 1 M Na_2_SO_4_ was adopted as the electrolyte. Generally, the Na_2_SO_4_ electrolyte presents a larger working voltage (−1.8–0 V) than KOH electrolyte (−1–0 V), and relatively low toxicity to the organic electrolyte. Fig. 9a exhibits CV curves of the assembled symmetrical supercapacitor at various working voltages between −1.2–0 and −2.0-0 V with a scan rate of 50 mV s^−1^. Consequently, the symmetrical supercapacitor can operate at −1.8 V, and the anode current did not clearly increase, meaning that the supercapacitor can stably function at −1.8–0 V.

Fig. 9b shows CV curves at various scanning rates between 5 and 100 mV s^−1^ with an operating voltage of −1.6–0 V. The GCD curves (Fig. 9c) showed triangular symmetry, and it was further proved that the main electrochemical behavior of the YPC-4 electrodes is that of an EDLC. Fig. 9d shows that the *C* values were 389.0, 239.1, 200.5, 151.9, and 93.8 F g^−1^ when the current density was 0.5, 1.0, 2.0, 5.0, and 10.0 A g^−1^ in 1 M Na_2_SO_4_ electrolyte, respectively.

In comparison to the *C* value in the KOH electrolyte, the capacitance of every YPC-4 electrode in neutral solution slightly decreased. We attributed the capacitance drop to the size effect of electrolyte ions. The size of hydrated Na^+^ (3.58 Å) in an aqueous solution exceeded that of K^+^ (3.31 Å), and the size of hydrated SO_4_^2−^ (3.79 Å) exceeded that of OH^−^ (3.00 Å) as well.^54^ Larger hydrated ions indicate lower mobility, which causes a reduction in capacitive performance.^55^

Fig. 9e displays the Nyquist plot for the YPC-4 monolithic electrode in Na_2_SO_4_ solution. The curve shape is similar to that measured in KOH electrolyte, implying that the electrolyte ion transfer in both electrolytes is fast. Furthermore, the device possessed a high specific energy of 34.6 W h kg^−1^ when the specific power was 200.1 W kg^−1^ and a praiseworthy specific energy of 8.3 W h kg^−1^ when the specific power was 4000.0 W kg^−1^ (Fig. 9f). In the two-electrode system, these proven electrochemical properties indicated that the hierarchical porous carbon samples prepared from yam waste are a potential energy conversion and storage material that can be used to construct supercapacitors.

## 4. Conclusion

O–N co-doped graded porous carbon materials were prepared by a simple carbonization and KOH activation of yam waste. The product not only exhibited an ultra-high SSA of 2382 m^2^ g^−1^, but also presented the most optimal pore distribution and appropriate N and O heteroatom doping. These advantages provide excellent energy storage properties for the YPC-*n* electrodes, with a remarkable *C* of 423.23 F g^−1^ in the three-electrode system when the current density was 0.5 A g^−1^, which is among the highest capacitance ever reported based on biomass carbon materials.

The results also revealed a high rate capability (68% capacitance retention at 10 A g^−1^) and large cycle stability (3.6% loss over 10 000 cycles). Moreover, in 6 M KOH electrolyte, the assembled symmetrical supercapacitor provided a large *C* of 387.3 F g^−1^ at 0.5 A g^−1^. It also presented a high specific energy of 34.6 W h kg^−1^ when the specific power was 200.1 W kg^−1^ and an impressive specific energy of 8.3 W h kg^−1^ when the specific power was 4000.0 W kg^−1^ in 1 M Na_2_SO_4_ electrolyte.

These excellent electrochemical properties indicate that the graded porous carbon samples prepared from yam waste are potential energy conversion and storage materials. The results provide data that can be used in this expanding and increasingly vital research area for the green synthesis of starch biomass materials for energy storage.

## Conflicts of interest

There are no conflicts to declare.

## Supplementary Material

RA-011-D1RA06154B-s001
