# Supplementary material for: Nitrogen and oxygen Co-doped porous carbon derived from yam waste for high-performance supercapacitors
Source: RSC Adv. 2021 Oct 8;11(53):33208–18. doi: 10.1039/d1ra06154b (PMC9042292; doi:10.1039/d1ra06154b)
Supplement: RA-011-D1RA06154B-s001 [file RA-011-D1RA06154B-s001.pdf]

Electronic Supplementary Material (ESI) for RSC Advances.
This journal is © The Royal Society of Chemistry 2021

Supplementary Information

Nitrogen and Oxygen Co-doped Porous Carbon Derived from Waste Yam for High-Performance Supercapacitors

Zhaojin Li[†], *Qian Liu*[†], *Lizhi Sun*[†], *Ning Li*[‡], *Xiaofeng Wang*[‡], *Qiujun Wang*[†], *Di Zhang*[†,*], *Bo Wang*[†,*]

[†] Hebei Key Laboratory of Flexible Functional Materials, School of Materials Science and Engineering, Hebei University of Science and Technology, Hebei 050018 (China)

[‡] Shenzhou Engineering Plastics Company Limited, Boling East Road 106, Shenzhou Economic Development Zone, Hebei 053800 (China)

* Author to whom correspondence should be addressed: School of Materials Science and Engineering，Hebei University of Science and Technology, Hebei 050018 (China)

E-mail addresses：wangbo1996@gmail.com, zd1210207@163.com

Table S1. The organic element contents in the YPC-4 sample.

YPC-4				
Elemental analysis	C (wt%)	H (wt%)	N (wt%)	O (wt%)
	44.89	2.83	0.33	46.42

YPC-4		
Elemental analysis	K (wt%)	Al (wt%)
	1.8	3.5

Table S2. The mass content of ash in the YPC-4 sample.

Table S3. Comparison of the capacitive performance of carbon materials in the literatures.

Materials	Activating Agent	Specific Surface Area (m^2 g^{-1})	Electrolyte	Current Density	Specific Capacitance ($F \cdot g^{-1}$)	Ref. No.
**Tea-waste**	KOH	1610	6 M KOH	1 A g^{-1}	332	1
**Loofah sponge**	KOH	2718	6 M KOH	1 A g^{-1}	309.6	2
**Coconut shell**	$ZnCl_2$	1874	6 M KOH	1 A g^{-1}	268	3
**Oil Palm Kernel Shell**	KOH	730	6 M KOH	0.5A g^{-1}	57.3	4
**Tree bark**	nano-ZnO	1511.91	6 M KOH	0.5 A g^{-1}	286	5
**Soybean**	KOH	1749	6 M KOH	0.5 A g^{-1}	243.2	6
**Albizia flowers**	KOH	2757.63	6 M KOH	0.5 A g^{-1}	406	7
**Cicada slough**	KOH	1676	6 M KOH	1 A g^{-1}	355	8
**Elm samara**	KOH	1947	6 M KOH	1 A g^{-1}	470	9
**straw**	KOH	1122	6 M KOH	1 A g^{-1}	337	10
**Starch**	MgO	2300	6 M KOH	1 A g^{-1}	229	11
**Waste potato peel**	KOH	1911.5	1 M Na_2SO_4	1 A g^{-1}	323	12
**Rotten potatoes**	KOH	960	6 M KOH	1 A g^{-1}	269	13
**This work**	KOH	2382	6 M KOH	0.5 A g^{-1}	423	-

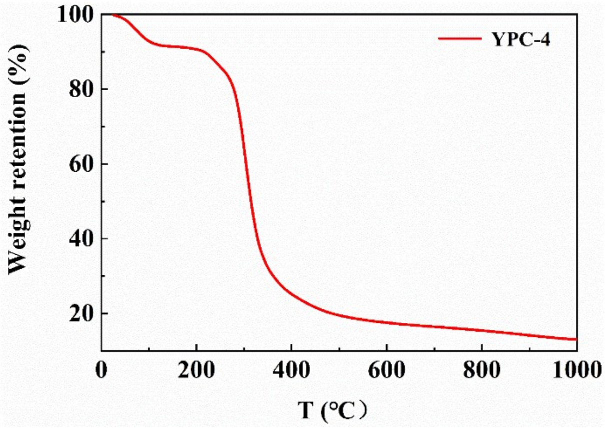

Figure S1. Thermogravimetric curve of YPC-4 sample.

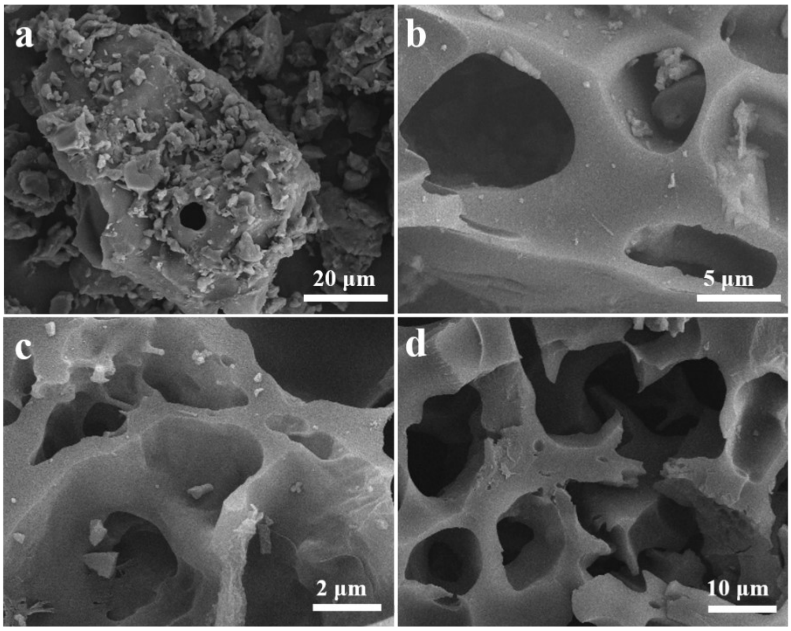

Figure S2. Morphology of the samples before and after activation. (a) SEM image of the YPC-4 sample before activation; (b) SEM image of the YPC-2 sample after activation; (c) SEM image of the YPC-3 sample after activation; (b) SEM image of the YPC-5 sample after activation.

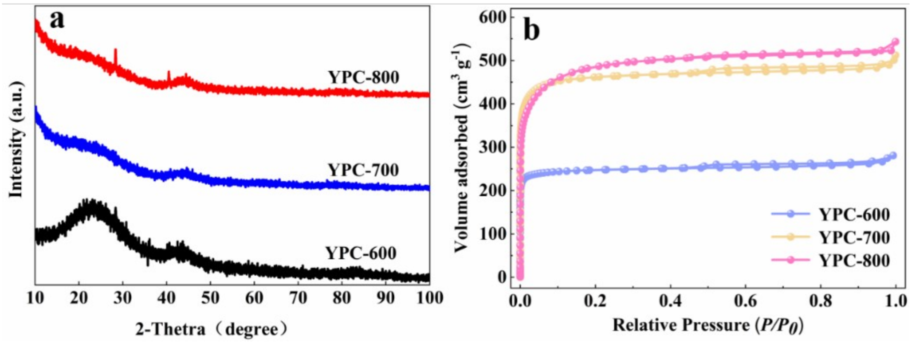

Figure S3. (a) XRD patterns and (b) N_2 adsorption-desorption isotherms of the YPC-600, YPC-700, and YPC-800 samples.

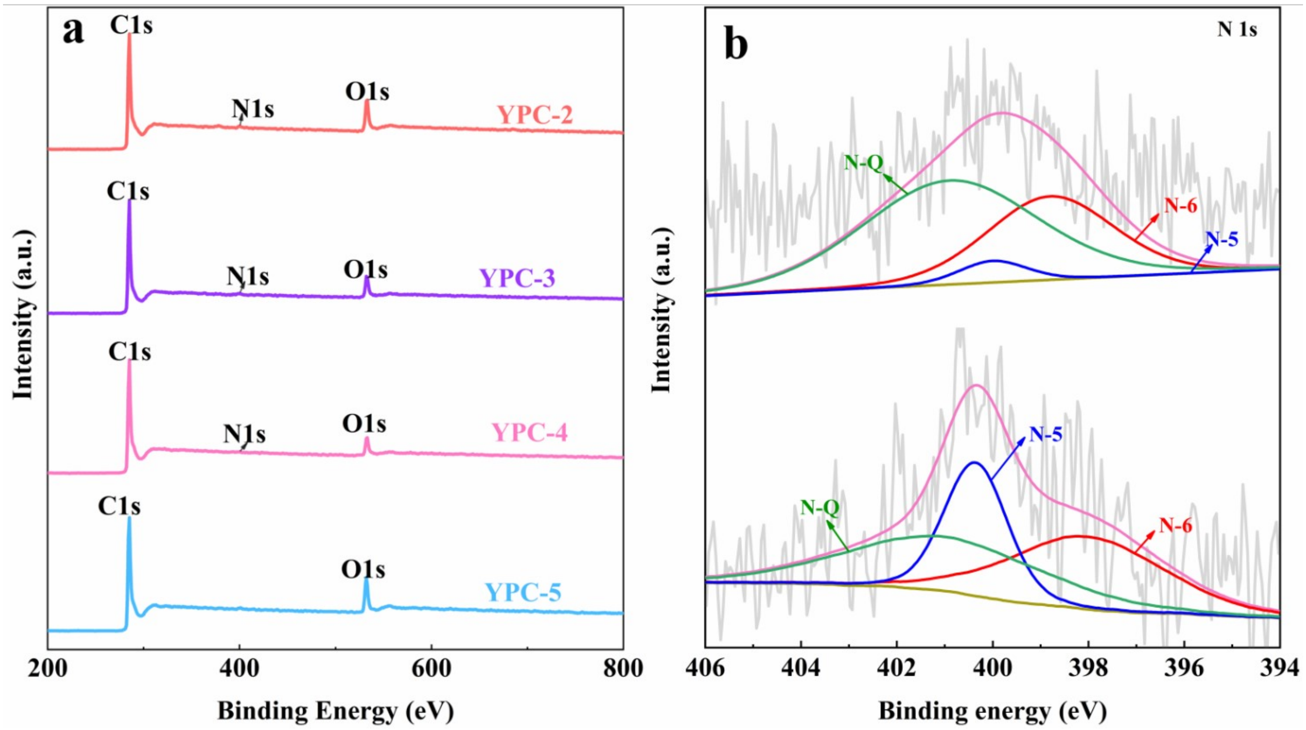

Figure S4. XPS spectra of (a) the YPC-2, YPC-3, YPC-4, and YPC-5 samples and (b) N 1s for YPC-2, YPC-3 samples.

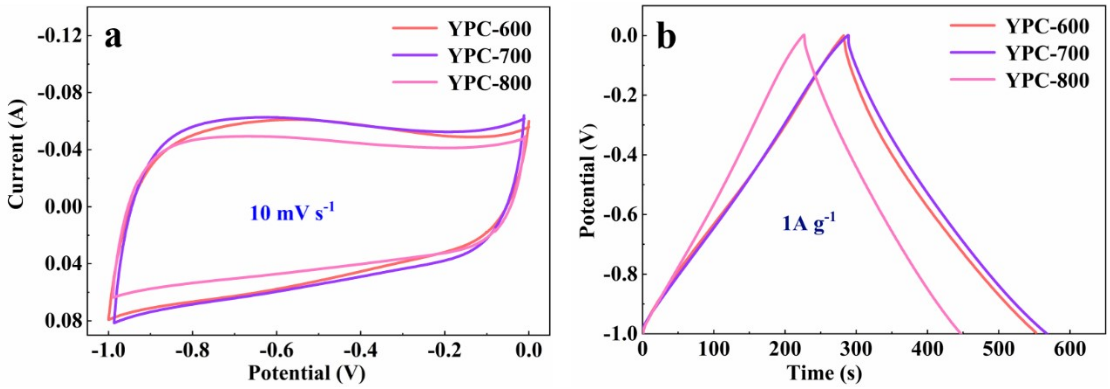

Figure S5. Electrochemical performance of the YPC-600, YPC-700 and YPC-800 samples in three-electrode system. (a) CV curves at 10 $mV{\cdot}s^{-1}$; (b) GCD profiles at 1 $A{\cdot}g^{-1}$.

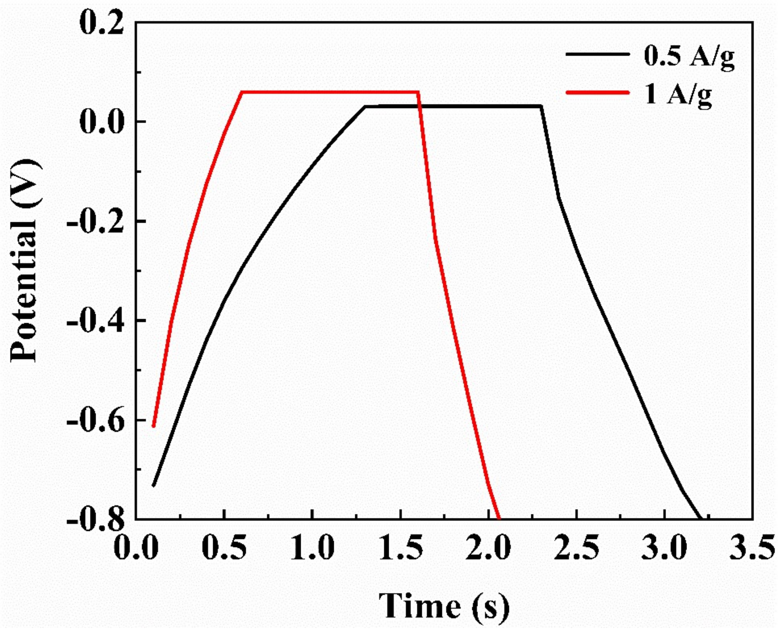

Figure S6. The GCD curves with ash as the electrode materials.

References

1 A. Khan, R. A. Senthil, J. Pan, S. Osman, Y. Sun and X. Shu, Electrochim. Acta, 2020, 335, 135588.

2 X. L. Su, J. R. Chen, G. P. Zheng, J. H. Yang, X. X. Guan, P. Liu and X. C. Zheng, Appl. Surf. Sci., 2018, 436, 327–336.

3 L. Sun, C. Tian, M. Li, X. Meng, L. Wang, R. Wang, J. Yin and H. Fu, J. Mater. Chem. A, 2013, 1, 6462–6470.

4 I. I. Misnon, N. K. M. Zain and R. Jose, Waste and Biomass Valorization, 2019, 10, 1731–1740.

5 F. Yu, Z. Ye, W. Chen, Q. Wang, H. Wang, H. Zhang and C. Peng, Appl. Surf. Sci., 2020, 507, 145190.

6 G. Lin, R. Ma, Y. Zhou, Q. Liu, X. Dong and J. Wang, Electrochim. Acta, 2018, 261, 49–57.

7 F. Wu, J. Gao, X. Zhai, M. Xie, Y. Sun, H. Kang, Q. Tian and H. Qiu, Carbon N. Y., 2019, 147, 242–251.

8 H. Jia, J. Sun, X. Xie, K. Yin and L. Sun, Carbon N. Y., 2019, 143, 309–317.

9 C. Chen, D. Yu, G. Zhao, B. Du, W. Tang, L. Sun, Y. Sun, F. Besenbacher and M. Yu, Nano Energy, 2016, 27, 377–389.

10 S. Liu, Y. Zhao, B. Zhang, H. Xia, J. Zhou, W. Xie and H. Li, J. Power Sources, 2018, 381, 116–126.

11 J. Cao, C. Zhu, Y. Aoki and H. Habazaki, ACS Sustain. Chem. Eng., 2018, 6, 7292–7303.

12 D. Khalafallah, X. Quan, C. Ouyang, M. Zhi and Z. Hong, Renew. Energy, 2021, 170, 60–71.

13 X. Chen, K. Wu, B. Gao, Q. Xiao, J. Kong, Q. Xiong, X. Peng, X. Zhang and J. Fu, Waste and Biomass Valorization, 2016, 7, 551–557.
